# Lower potassium intake is associated with increased wave reflection in young healthy adults

**DOI:** 10.1186/1475-2891-13-39

**Published:** 2014-04-28

**Authors:** Shannon Lennon-Edwards, Brittany R Allman, Taylor A Schellhardt, Courtney R Ferreira, William B Farquhar, David G Edwards

**Affiliations:** 1 Department of Behavioral Health and Nutrition, University of Delaware, 25 North College Avenue, Newark, DE 19716, U.S.A; 2 Department of Kinesiology and Applied Physiology, University of Delaware, Newark, DE, U.S.A

**Keywords:** Potassium, Sodium, Wave reflection, Arterial stiffness

## Abstract

**Background:**

Increased potassium intake has been shown to lower blood pressure (BP) even in the presence of high sodium consumption however the role of dietary potassium on vascular function has received less attention. The aim of this study was to evaluate the relationship between habitual intake of sodium (Na) and potassium (K) and measures of arterial stiffness and wave reflection.

**Methods:**

Thirty-six young healthy adults (21 M, 15 F; 24 ± 0.6 yrs; systolic BP 117 ± 2; diastolic BP 63 ± 1 mmHg) recorded their dietary intake for 3 days and collected their urine for 24 hours on the 3rd day. Carotid-femoral pulse wave velocity (PWV) and the synthesis of a central aortic pressure waveform (by radial artery applanation tonometry and generalized transfer function) were performed. Aortic augmentation index (AI), an index of wave reflection, was calculated from the aortic pressure waveform.

**Results:**

Subjects consumed an average of 2244 kcals, 3763 mg Na, and 2876 mg of K. Average urinary K excretion was 67 ± 5.3 mmol/24 hr, Na excretion was 157 ± 11 mmol/24 hr and the average Na/K excretion ratio was 2.7 ± 0.2. An inverse relationship between AI and K excretion was found (r = -0.323; p < 0.05). A positive relationship between AI and the Na/K excretion ratio was seen (r = 0.318; p < 0.05) while no relationship was noted with Na excretion alone (r = 0.071; p > 0.05). Reflection magnitude, the ratio of reflected and forward waves, was significantly associated with the Na/K excretion ratio (r = 0.365; p <0.05) but not Na or K alone. PWV did not correlate with Na or the Na/K excretion ratio (p > 0.05) but showed an inverse relationship with K excretion (r = -0.308; p < 0.05).

**Conclusions:**

These data suggest that lower potassium intakes are associated with greater wave reflection and stiffer arteries in young healthy adults.

## Background

While excess dietary sodium intake is associated with the development of cardiovascular disease [1,2], increased dietary potassium intake has been shown to be protective [3,4]. Given the high dietary levels of sodium and low potassium habitually consumed by the average person [5], understanding the cardiovascular effects of a high sodium/low potassium diet has important clinical and public health implications.

Low sodium and high potassium diets have been shown to lower BP [3,6] however the relationship between sodium and potassium and vascular function has received much less attention. Increased arterial stiffness and wave reflection are independent predictors of cardiovascular events [7-11]. Acutely, a high sodium meal has been shown to increase augmentation index in normotensive adults [12] while two weeks of a high salt intake in young, healthy, normotensive males increased wave reflection and carotid BP [13]. Further, two weeks of dietary sodium restriction improved carotid arterial compliance and augmentation index in older individuals with systolic hypertension [14]. Only recently has the role of potassium on vascular function been explored with most studies focused on prehypertension and hypertension [15,16]. The results are inconsistent, as some have shown no change in arterial stiffness [15] while others have shown reduced arterial stiffness with potassium supplementation [16]. The interaction of sodium and potassium has gained much interest as several large trials have demonstrated that a high urinary excretion ratio may play a role in the development of hypertension and other forms of cardiovascular disease [17,18]. Arterial stiffening begins at a young age and may be accelerated due to lifestyle choices [19-21] therefore exploring the relationship between dietary intake of sodium and potassium and vascular health in young healthy adults is important but has not been explored.

The purpose of the present study was to determine whether habitual intake of dietary sodium and potassium, as assessed by urinary excretion, is associated with arterial stiffness and wave reflection in healthy, young adults. This was assessed by carotid to femoral PWV and pulse wave analysis of the central aortic pressure wave. We hypothesized that indices of wave reflection and arterial stiffness would be positively associated with the urinary sodium to potassium excretion ratio. To test this hypothesis, we performed a cross-sectional study of healthy young adults in which they recorded their dietary intake for 3 days and collected their urine for 24 hours prior to vascular assessment.

## Methods

### Subjects

Thirty-six, healthy young subjects participated in this study (24 ± 0.6 yrs). All subjects were normotensive and free of disease. Exclusion criteria included obesity [BMI > 30 kg/m^2^], smoking, current use of cardiovascular and/or hypertensive medication and a history of hypertension, cardiovascular disease, malignancy, diabetes mellitus, or renal impairment. All women were studied in the early follicular phase of the menstrual cycle. Informed consent was obtained from all subjects, and the study protocol and procedures were approved by the Institutional Review Board of the University of Delaware and conform to the provisions of the *Declaration of Helsinki*.

### Experimental protocol

All subjects provided a complete medical history during the screening visit. A resting BP (GE Medical Systems, Dinamap Dash 2000, Milwaukee, WI), height and weight (Healthometer Scale, Continental Scale, Bridgeview, IL) were determined during this visit.

Following the screening visit, subjects were instructed and provided a packet with information on how to record their dietary intake for 3 days. Subjects were provided information on portion size estimation, instructed to eat as they normally would, and to include two weekdays and one weekend day in their record. The last day of diet recording preceded their visit to the laboratory. Completed diet records were reviewed at their next visit to the laboratory. Each diet record was analyzed for total energy, fat, protein, carbohydrate, sodium, and potassium content, as well as the other micronutrients using Nutrition Data System for Research (NDSR; Minneapolis, MN).

Urine was collected on the last day of diet recording for 24-hours and kept in a cool dark container. All subjects kept a urine collection log and marked the time of each collection to document that the urine was collected. The total volume, urinary electrolytes (EasyElectrolyte Analyzer, Medica, Bedford, MA), and urine osmolality (Advanced 3D3 Osmometer, Advanced Instruments, Norwood, MA) were assessed from an aliquot of the 24-hour collection period. Free water clearance and fractional excretion of sodium, potassium and chloride were calculated using standard equations.

Physical activity of each subject was tracked by use of an Actical device (Respironics, Phillips Electronics). The Actical device provides a measure of energy expenditure above rest. Subjects wore the device on their right hip on days coinciding with the three-day diet record. Activity levels were assessed using the Actical Software (Version 2.12) and ActiReader (Respironics, Phillips Electronics).

### Pulse wave analysis

Subjects were asked not to eat for 4 hours, consume alcohol or caffeine for 12 hours, and exercise for 24 hours prior to testing. Time of testing was based on subject availability. All measurements were made in the supine position at an ambient temperature of 20-21°C.

Applanation tonometry was used to record a radial arterial waveform by placing a high-fidelity strain-gauge transducer over the radial artery (Millar Instruments). The radial waveform was calibrated from the brachial sphymomanometric measurement of systolic and diastolic pressure (GE Medical Systems, Dinamap Dash 2000, Milwaukee, WI). A central aortic pressure wave was synthesized from the measured radial artery pressure waveform with the SphygmoCor Px system (AtCor Medical, Sydney, Australia), which uses a transfer function and is FDA approved. Central pressures and augmentation index (AI) were obtained from the synthesized wave. AI is an index of wave reflection and is influenced by arterial stiffness. AI is defined as the ratio of reflected wave amplitude and pulse pressure, or AI = (P_s_ - P_i_)/(P_s_ - P_d_), where P_s_ is peak systolic pressure, P_d_ is end-diastolic pressure, and Pi is an inflection point marking the beginning upstroke of the reflected pressure wave. The travel time (T_R_) of the forward wave from the heart to the major reflecting site and back was measured from P_d_ to P_i_. Wave separation analysis was performed using the SphygmoCor software (version 9) on the central pressure waveform to determine forward and reflected wave components using a modified triangular flow waveform [22]. Reflection magnitude (RM) was calculated as the ratio of the amplitudes of reflected/forward waves. RM allows for assessment of reflected wave amplitude that is not influenced by timing of the reflected wave, HR, and height that confound AI [23].

### Pulse wave velocity

Carotid-femoral PWV was measured using tonometry to record both carotid artery and femoral artery waveforms simultaneously while the subject was at rest in a supine position. External distances were measured proximally from the carotid measurement site to the sternal notch, and distally from the sternal notch to the umbilicus and from the umbilicus to the femoral measurement site. The distance from the carotid to the sternal notch was subtracted from the sternal notch to femoral measurement. Carotid-femoral PWV was calculated by dividing the measured aortic distance (distal – proximal) by the average measured time delay between the initial upstrokes of twelve consecutive corresponding carotid and femoral waveforms.

### Blood analyses

A venous blood sample was used to measure hemoglobin (Hb 201+ model, HemoCue, Lake Forest, CA), hematocrit (Clay Adams Brand, Readacrit® Centrifuge, Becton Dickinson, Sparks, MD), serum electrolytes (EasyElectrolyte Analyzer, Medica, Bedford, MA), and plasma osmolality (Advanced 3D3 Osmometer, Advanced Instruments, Norwood, MA).

### Statistical analyses

The purpose of the present study was to determine whether habitual intake of dietary sodium and potassium, as assessed by urinary excretion, is associated with arterial stiffness and wave reflection. Therefore, Pearson correlations were performed to determine the relationships between measures of habitual intake of sodium and potassium (urinary excretion of sodium and potassium and the sodium/potassium excretion ratio) and arterial stiffness (PWV) and indices of wave reflection (AI, Tr, RM). Additionally, correlations were determined between measures of habitual intake of sodium and potassium with blood pressure and heart rate that are known to influence stiffness and wave reflection. Because physical activity can influence arterial stiffness and wave reflection we examined relationships between it and PWV, AI and BP. Partial correlations were performed to control for height, HR, and MAP when significant correlations were found with AI. Lastly, unpaired t-tests were performed to compare variables between males and females. ANCOVA was also used to compare AI between men and women with height as the covariate. Data are presented as means and standard error of mean. Statistical significance was set at p < 0.05. A sample size of 36 subjects provided us 80% power to detect a relationship of r = 0.38 at an alpha level of 0.05.

## Results

Subject characteristics are presented in Table 1. As shown, all subjects had a BMI in the normal range and normal iron status and electrolyte levels. Physical activity was assessed and on average subjects expended 1025 ± 279 kcalories/day above resting energy expenditure. Physical activity did not correlate significantly with PWV, AI, or  %BP (p > 0.05). Hemodynamic variables are presented in Table 2. Average peripheral BPs was 117 ± 2/63 ± 1 mm Hg. Female subjects did have a lower systolic BP and PP (p < 0.05). There was no correlation between HR or measures of BP and AI or PWV.

**Table 1:** Subject characteristics

	**All subjects**	**Males**	**Females**
*Demographic data*		(n = 21)	(n = 15)
Age (yr)	24 ± 0.6	24 ± 0.7	23 ± 0.9
Height (cm)	174 ± 1.6	179.4 ± 1.5	166.2 ± 1.7*
Mass (kg)	73.4 ± 1.9	79.6 ± 2.0	65 ± 1.8*
BMI (kg/m^2^)	24.2 ± 0.5	24.7 ± 0.6	23.5 ± 0.7
*Biochemical parameters*			
Hemoglobin (mg/dL)	14.5 ± 0.2	15.2 ± 0.7	13.4 ± 0.2*
Hematocrit (%)	41.4 ± 0.7	43.5 ± 0.7	38.4 ± 1.0*
Serum sodium (mmol/L)	137.6 ± 0.3	137.6 ± 0.4	137.5 ± 0.7
Serum potassium (mmol/L)	4.2 ± 0.01	4.2 ± 0.06	4.1 ± 0.1
Serum chloride (mmol/L)	103 ± 0.4	102.9 ± 0.4	103.2 ± 0.5
Plasma osmolality (mOsm/kg H_2_O)	287 ± 0.7	287.3 ± 0.7	286.8 ± 0.9

Values are mean ± SE. BMI, body mass index. *P < 0.05 vs. males.

**Table 2:** Hemodynamic data

	**All subjects**	**Males**	**Females**
*Peripheral BP*			
Systolic pressure (mm Hg)	117 ± 2	121 ± 2	112 ± 3*
Diastolic pressure (mm Hg)	63 ± 1	64 ± 2	63 ± 2
MAP (mm Hg)	80 ± 1	80.3 ± 2	79 ± 3
PP (mm Hg)	54 ± 2	58 ± 2	49 ± 2*
*Central BP*			
Systolic BP (mm Hg)	98 ± 2	99 ± 2	95 ± 3
Diastolic BP (mm Hg)	64 ± 1	64 ± 2	64 ± 2
MAP (mm Hg)	80 ± 1	79 ± 2	79 ± 3
PP (mm Hg)	34 ± 1	35 ± 2	31 ± 2*
Heart rate (bpm)	57 ± 2	56 ± 3	61 ± 3
Tr (ms)	167 ± 3.9	170.1 ± 5.0	162.4 ± 6.0
Ejection duration (ms)	338 ± 3.5	333.5 ± 4.1	343 ± 6.0
Augmentation index (%)	2.2 ± 1.6	-1.1 ± 2.1	6.9 ± 2.2*
Forward wave amplitude (mmHg)	30.4 ± 1.2	32.1 ± 1.6	28.6 ± 1.8
Reflected wave amplitude (mmHg)	13.4 ± 0.5	13.1 ± 0.7	13.8 ± 0.7
Reflection magnitude (%)	44.6 ± 1.4	40.8 ± 1.5	48.6 ± 2.2*
Carotid-femoral PWV (m/s)	5.2 ± 0.2	5.2 ± 0.4	5.2 ± 0.3

Values are mean ± SE. BP, blood pressure; MAP, mean arterial pressure; PP, pulse pressure; Tr, time delay of the reflected wave; PWV, pulse wave velocity. *P < 0.05 vs. males.

### Dietary intake

Table 3 presents the habitual intake of the subjects over the 3 days recorded. Subjects consumed on average approximately 2200 (2244 ± 123) kcalories/day with approximately 50% of this energy coming from carbohydrates, 31% from fat, and 18% from protein, all falling within the acceptable macronutrient distribution ranges recommended by the Dietary Reference Intakes [24]. As expected, females had lower energy consumption (p < 0.05). Sodium intake averaged 3763 ± 286 mg/day and was therefore significantly above recommended levels set by the 2010 American Dietary Guidelines an [25]. However, there was a wide range of consumption in this group (1410 – 9026 mg). Further, males consumed significantly more sodium than females (p < 0.05). On the other hand, potassium intake was below the recommended guidelines of 4700 mg at 2876 ± 161 mg/day however there was a wide range of intake as well (1227 to 4920 mg). In this regard, there was no difference between males and females in their potassium consumption. The dietary intake data of both sodium and potassium significantly correlated with their respective urinary excretion data (potassium: r = 0.52, p < 0.001; sodium: r = 0.47, p < 0.005) indicating that excretion reflects dietary intake. Urinary excretion values are shown in Table 4. Calcium recommendations were met while magnesium intake was slightly low for both males and females. The sodium to potassium intake ratio of 1.4 ± 0.1 is above the recommended ratio of 0.49 (2300 mg Na/4700 mg K) based on the 2010 Dietary Guidelines for Americans for these nutrients.

**Table 3:** Diet characteristics

**Nutrient**	**All subjects**	**Males**	**Females**
Total energy (kcals/d)	2244 ± 123	2492 ± 167	1798 ± 106*
CHO (g/d)	278 ± 18	317 ± 24.6	216 ± 16.7*
FAT (g/d)	77.5 ± 5	87.5 ± 6.6	63 ± 5.4*
PTN (g/d)	97 ± 6.0	102.3 ± 5.7	89.6 ± 13.9
Sodium (mg/d)	3763 ± 286	4212 ± 409	2879 ± 213*
Potassium (mg/d)	2876 ± 161	2967 ± 201	2629 ± 289
Calcium (mg/d)	990.7 ± 74	1040.7 ± 95	917 ± 125
Magnesium (mg/d)	342.6 ± 26	373 ± 35	289 ± 38
Phosphorus (mg/d)	1422 ± 83	1538 ± 98	1237 ± 143
Caffeine (mg/d)	109.3 ± 18	78.7 ± 18.1	155 ± 37*
Alcohol (g/d)	8.7 ± 2.6	11.3 ± 4.1	5.2 ± 1.9
Sodium/potassium intake ratio	1.4 ± 0.1	1.5 ± 0.14	1.1 ± 0.2

Values are mean ± SE.*P < 0.05 vs. males.

**Table 4:** Urine excretion data

**Urinary markers**	**All subjects**	**Males**	**Females**
Urine osmolality (mOsm/kg H_2_O)	563 ± 38	612 ± 49	494 ± 58
24-hour sodium excretion (mmol/24 hr)	157 ± 11	163.2 ± 16.4	148.6 ± 13
24-hour potassium excretion (mmol/24 hr)	67 ± 5.3	65.8 ± 5.7	68.4 ± 10
24-hour sodium/potassium excretion ratio	2.7 ± 0.2	2.67 ± 0.3	2.83 ± 0.4

Values are mean ± SE.

### Wave reflection and arterial stiffness

**Figure 1:**
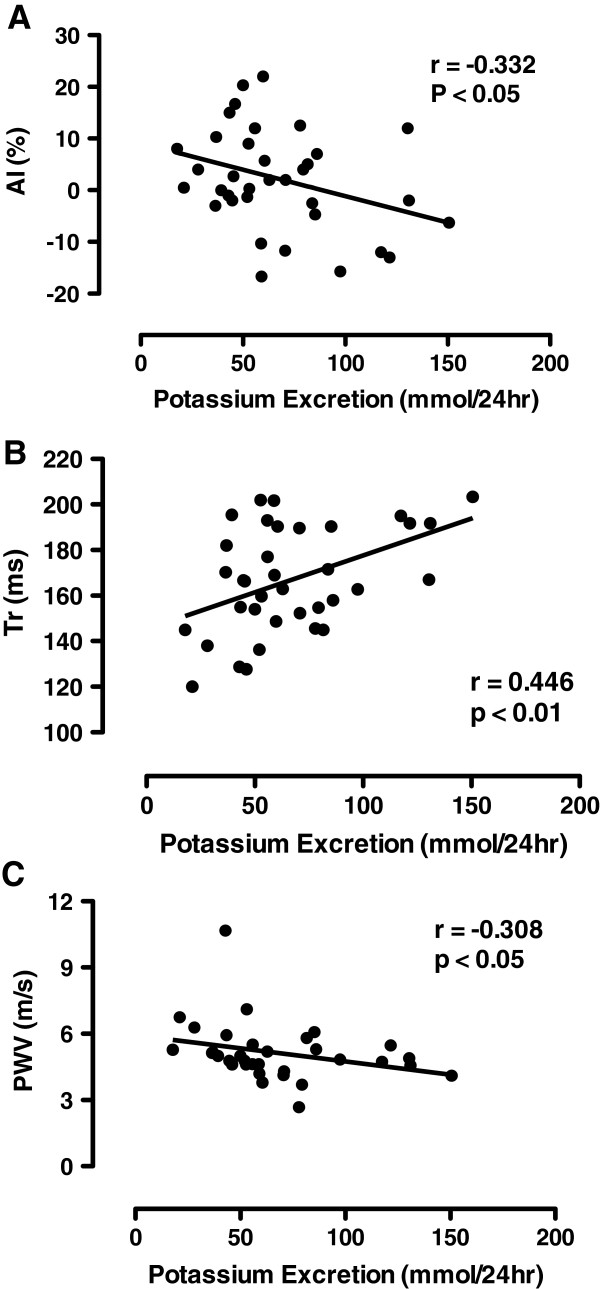
**Pearson correlations between urinary excretion of potassium and parameters of pulse wave analysis. (A)** Augmentation index (AI) **(B)** Time delay of the reflected wave (Tr); and **(C)** pulse wave velocity (PWV).

Average AI was 2.2% ± 1.6 for all subjects and PWV was 5.2 ± 0.3 m/s. In comparing the sexes, AI was higher in females compared to males (p < 0.05), however when corrected for height, this difference no longer existed. There was no relationship between sodium excretion and AI (r = 0.07, p > 0.05). A significant inverse correlation was found between potassium excretion and AI (Figure 1A) suggesting that those subjects with lower potassium intakes had greater wave reflection (r = -0.332; p < 0.05). The relationship between the sodium/potassium excretion ratio and AI was also significant (r = 0.318; p < 0.05). Further, Tr was significantly correlated with potassium excretion (r = 0.446; p < 0.01, Figure 1B) suggesting that those with a lower intake of potassium also exhibited a shorter time delay of the reflected wave. The relationship between potassium excretion and PWV suggests that those with greater potassium intake have a slower velocity (r = -0.308; p < 0.05) (Figure 1C) however this was not seen between the sodium/potassium excretion ratio and PWV (r = 0.104; p > 0.05). Further, males and females had a similar PWV and Tr.

AI can be influenced by height, HR, and MAP so partial correlations were performed to control for these variables. AI remained significantly associated with both potassium excretion and the sodium/potassium excretion ratio when controlling for height (potassium: r = -0.323, p <0.05; ratio: r = 0.324, p <0.05). When HR and MAP were added to the relationship between AI and the ratio, this remained significant (r = 0.303, p <0.05) however the relationship between AI and potassium was no longer significant (r = -0.265, p = 0.068). RM, the ratio of reflected and forward waves, was significantly associated with the sodium/potassium excretion ratio (r = 0.365; p <0.05) but not sodium or potassium alone providing further support that both potassium and sodium intake may be important mediators of wave reflection.

## Discussion

The purpose of the present study was to determine if a relationship existed between habitual intake of sodium and potassium on arterial stiffness and wave reflection in healthy, young adults. Our data indicate that potassium had the greatest influence on wave reflection in this group of healthy, young adults. The primary novel finding of this study was that a lower potassium excretion was associated with greater wave reflection and a longer time until the return of the reflected wave. Further, pulse wave velocity is faster in those with lower potassium intake. Sodium excretion alone was not associated with AI however the sodium/potassium excretion ratio was indicating that the effect of diet on wave reflection appears to have been driven primarily by potassium intake in this group of subjects. These findings are novel in that potassium has primarily been associated with its BP lowering effects [3,4,26,27]. Similar to our results, other data suggest that urinary sodium is not associated with AI as well [28]. AI is an important measure of wave reflection in that it represents the combined effects of reflected wave amplitude and timing as well as left ventricular function [23]. RM, determined by wave separation analysis, is not influenced by overlap of forward and reflected waves [23] and was also related to the sodium/potassium excretion ratio but not sodium or potassium excretion alone. These findings provide further support that intake of both potassium and sodium may be important in influencing wave reflection.

To our knowledge, the present study is the first to examine habitual intake of potassium on wave reflection. To date, investigations of intake of both sodium and potassium have primarily focused on their impact on BP. Studies such as the DASH diet trial [3], the ENCORE study [20] and those focusing on dietary patterns [26] have provided evidence for potassium’s BP lowering effects. A meta-analysis by Whelton et al. [4] demonstrated that a low potassium intake plays a role in elevated BP. While it appears potassium has beneficial effects on BP it may also have positive vascular effects. The China Salt Substitute study compared the effects of a salt substitute containing 25% potassium chloride to regular salt in subjects with significant risk factors for vascular disease over a 12-month period [29]. They found that the salt substitute increased time delay of the reflected wave but resulted in no change in AI. In a group of subjects similar to our sample, a study by Matthesen et al., [30] examined the impact of supplementation of 100 mmol potassium chloride daily for 28 days. Subjects consumed a standardized diet for 4 days prior to testing which contained either 150 or 200 mmol of sodium depending on energy needs. A small but significant increase in PWV was observed without a change in AI or BP in this group of young healthy adults. The observed increase in PWV in response to potassium supplementation correlated with increased water absorption and aldosterone concentration. It is unclear why potassium caused PWV to increase but it should be noted that this was observed with supplementation, not consumption of whole foods. Berry et al. [15] supplemented pre-hypertensive subjects for 6 weeks with 40 mmol potassium citrate or increased fruit and vegetable consumption, providing either 20 or 40 mmol of additional potassium. They found no change in PWV or AI following 6 weeks of supplementation. It may be that the potassium supplementation was not great enough in this study population as even the DASH diet alone provides roughly 45 mmol of potassium in addition to being low in sodium. Further, the amount of potassium in the background diet is not clear and may have influenced the results. A study of stage-1 hypertensive subjects taking 64 mmol of either potassium bicarbonate or potassium chloride for 4 weeks found significant decreases in PWV compared to placebo [16]. Finally, in a larger cross-sectional study, the sodium to potassium intake ratio was shown to be associated with AI and measures of arterial stiffness in a J-shaped manner [31] illustrating that both very high and low intake ratios correlate with increased arterial stiffness.

In the present study, a higher potassium excretion was associated with lower AI and a slower PWV. Thus, it is likely that arterial stiffness plays some role in the relationship observed between potassium excretion and wave reflection by speeding the return of the reflected wave. An additional potential mechanism by which dietary potassium may influence the vasculature is through maintenance or improvement in endothelial function however we did not assess endothelial function in the present study. Inhibition of basal nitric oxide (NO) synthesis has been shown to increase AI [32,33]. Cell culture studies have demonstrated that a physiological increase in potassium concentration in the culture medium increased NO production [34] and potassium supplementation has been shown to improve brachial artery flow mediated dilation in humans [16]. Hence, it is plausible that individuals consuming greater amounts of potassium in their diet have lower wave reflection as a result of better endothelial function however future studies are necessary.

Limitations of our study include the modest sample size and cross-sectional design. Although our design does not allow us to determine mechanisms, we demonstrated that habitual intake of potassium may be an important mediator of wave reflection. Future studies should focus on a controlled feeding design that would allow for greater elucidation of cause and effect.

## Conclusions

In summary, we have demonstrated an inverse relationship between habitual potassium intake, wave reflection, and arterial stiffness in young, healthy adults. This suggests that potassium has potential to affect the vasculature beyond its notable blood pressure lowering effects. This is important, as most studies have utilized potassium supplementation and not evaluated habitual dietary intake of potassium. AI and PWV are associated with a higher incidence of coronary artery disease in middle-aged and older individuals [7,11,35]. Therefore, increasing potassium in the diet has the potential to reduce cardiovascular risk. Future work should evaluate the habitual intake of potassium in middle-aged and older adults.

## Abbreviations

AI: Augmentation index; BMI: Body mass index; BP: Blood pressure; FMD: Flow-mediated dilation; K: Potassium; MAP: Mean arterial pressure; Na: Sodium; PP: Pulse pressure; PWV: Pulse wave velocity; RM: Reflection magnitude; Tr: Time delay of the reflected wave.

## Competing interests

The authors declare that they have no competing interests.

## Authors’ contributions

SLE participated in the design of the study, performed statistical analysis and drafted the manuscript. BA, TAS, and CF all contributed significantly to subject recruitment and data collection. WBF assisted with study design and drafting of the manuscript. DGE participated in study design, assisted with data analysis and drafting of the manuscript. All authors approved the final version of the manuscript.
